# Supplementary material for: Proteomic Analysis of a Rat Streptozotocin Model Shows Dysregulated Biological Pathways Implicated in Alzheimer’s Disease
Source: Int J Mol Sci. 2024 Feb 28;25(5):2772. doi: 10.3390/ijms25052772 (PMC10931650; doi:10.3390/ijms25052772)
Supplement: Supplementary file 1 [file ijms-25-02772-s001.zip › Supplementary.pdf]

Supplementary Information

Proteomic analysis of a rat streptozotocin model shows dysregulated biological pathways implicated in Alzheimer's Disease

Esdras Matheus Gomes da Silva, Juliana S. G. Fischer, Isadora de Lourdes Signorini Souza, Amanda Caroline Camillo Andrade, Paulo C. Carvalho, Ricardo Lehtonen Rodrigues Souza, Maria Aparecida Barbato Frazão Vital and Fabio Passetti

This file contains the description of:

- **Supplementary Tables S1-4**

Supplementary Tables

Supplementary Table S1. Proteins identified in the prefrontal cortex and hippocampus proteomes of the streptozotocin rat model and control (wild-type)

Supplementary Table S2. Peptides identified in the prefrontal cortex and hippocampus proteomes of the streptozotocin rat model and control (wild-type)

Supplementary Table S3. Protein quantification (normalized XIC) in the prefrontal cortex and hippocampus proteomes of the streptozotocin rat model and control (wild-type)

Supplementary Table S4. Fold change of proteins in the prefrontal cortex and hippocampus proteomes of the streptozotocin rat model and control (wild-type)
